# Digital environmental health: a digital platform for preliminary prevention and intervention

**DOI:** 10.4069/whn.2024.08.31

**Published:** 2024-09-30

**Authors:** SungChul Seo

**Affiliations:** Department of Nano, Chemical & Biological Engineering, College of Natural Science & Engineering, Seokyeong University, Seoul, Korea

## Introduction

In the mid-20th century, the world entered the era of digital technology, which has profoundly influenced every aspect of our daily lives, including work, learning, and social interactions [1]. The digital transformation began with binary computing systems and has evolved through various stages, including circuit chips, personal computers, the Internet, the World Wide Web, social media platforms, and smartphones [2]. During the early phases of this transformative period, international organizations, as well as regional and national governments, were among the first to devise strategies for adopting digital technologies and managing digital information. These plans primarily concentrated on enhancing digital capabilities within administrative and economic sectors.

The coronavirus disease 2019 pandemic in 2020 accelerated the pace of digitalization, leading to the widespread adoption of remote work, online learning, and e-commerce as lockdown measures were implemented globally [3]. Virtual meetings replaced physical ones, and digital wallets became more prevalent than cash. As digital systems became more compact, affordable, and diverse, their use shifted from a luxury to a necessity. Consequently, access to digital technology has become a social determinant of health, necessitating close examination and research from a health perspective [4].

### Digital environment

The term “digital environment” is typically defined as “the situation or space enabled by technology and digital devices transmitted through the Internet or other digital means” [5]. However, an alternative perspective suggests that the digital environment extends beyond digital materials since it has influenced non-digital media, imbuing them with digital characteristics due to their ubiquity and integration into daily life. Thus, the digital environment can be understood as encompassing the entire continuum, from the tangible aspects of computing devices, programming and information systems, and the network technologies that connect them, to the digital interfaces that result from human interactions.

### Digital environmental health

The field of public health known as environmental health examines the interactions between individuals and their surrounding environment [6]. This typically includes natural elements such as air, weather, water, chemicals, and radiation, as well as occupational hazards in work environments and built environments that encompass sanitation and hygiene facilities. Maintaining a balanced relationship with these environments is essential for promoting human health and well-being and for fostering sustainable, secure communities.

In recent years, there has been a growing global concern about the impact of digitalization on health, prompting numerous studies across the health sector. However, these studies often occur in silos, with experts working in isolation on specialized topics. The evaluation of digital systems in healthcare falls under health system management, while the effectiveness of digital technologies is explored in epidemiology and specific medical fields. Research in psychology has also concentrated on the effects of digital device usage and exposure on mental health and child development [7]. The wide-ranging effects of digitization, fueled by its widespread availability and accessibility, are being investigated not only in healthcare but also in fields such as economics, business, political governance, and social sciences. It is crucial for environmental health studies to thoroughly examine how digitalization changes our perceptions of the environment as a determinant of health and to explore the health impacts of the digital world.

### Digital health

Digital health employs information and communication technologies to effectively and accurately tackle a range of health challenges encountered by the general public [8]. This approach typically involves collecting data on individual health status, analyzing this information for preclinical or clinical assessments, and providing personalized interventions or monitoring for specific individuals or communities. As a result, a variety of tools have been developed, such as smartphones, mobile applications, wearable devices, personal health records, electronic medical records, and telemedicine.

Advances in digital health have broadened access to healthcare services, empowered patients to make informed decisions, enhanced patient education, and improved medication adherence [9]. Nonetheless, data from digital health monitoring devices can lead to adverse outcomes, such as diagnostic errors or treatment delays, due to the complexities involved in interpreting the data.

### Digital platforms

**Figure 1. f1-whn-2024-08-31:**
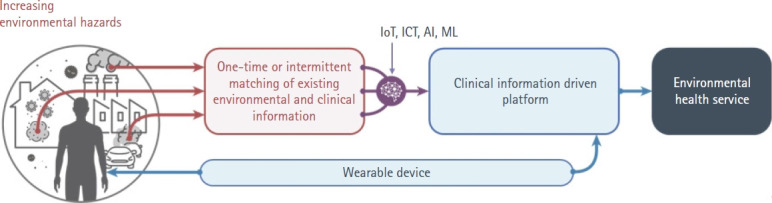
Conceptual flowchart of a digital device-based health prevention strategy based on environmental exposures. IoT: internet of things; ICT: information and communication technology; AI: artificial intelligence; ML: machine learning. Adated from the book of Ko et al. [10] with appropriate permission.

Exposure to environmental hazards has become increasingly complex, including particulate matter (PM), household chemicals, and biocides. This diversity complicates the collection of adequate data for assessing health effects, as variations in exposure receptors, duration, and intensity must be considered. The gap between the data produced by fourth-industrial technologies, such as the internet of things (IoT) and artificial intelligence (AI), and the information on environmental hazards continues to grow. Additionally, clinical data from hospital treatments and self-reported surveys are often outdated and unsuitable for secondary prevention efforts aimed at alleviating worsening disease symptoms. Therefore, it is necessary to obtain real-time data on environmental hazard concentrations and health information specific to exposure receptors. Aligning these datasets is crucial for a proactive approach to mitigating disease symptoms (Figure 1 [8]).

## The necessity of developing health impact assessment methods for environmental hazards

Conducting health impact assessments for residents in environmentally vulnerable areas, such as those near industrial complexes, thermal power plants, and limestone mines, is a critical investigative task. This task is mandated by *the Environmental Health Act* and overseen by the Ministry of Environment. However, these assessments, which are conducted annually or quarterly, yield limited clinical data in comparison to the extensive information on environmental hazard exposure. This imbalance restricts the ability to perform adequate and rational health assessments. Despite the significant costs associated with these analyses, traditional health impact evaluations are inefficient for assessing the long-term health effects of environmental changes due to their reliance on one-time studies of biological samples.

**Figure 2. f2-whn-2024-08-31:**
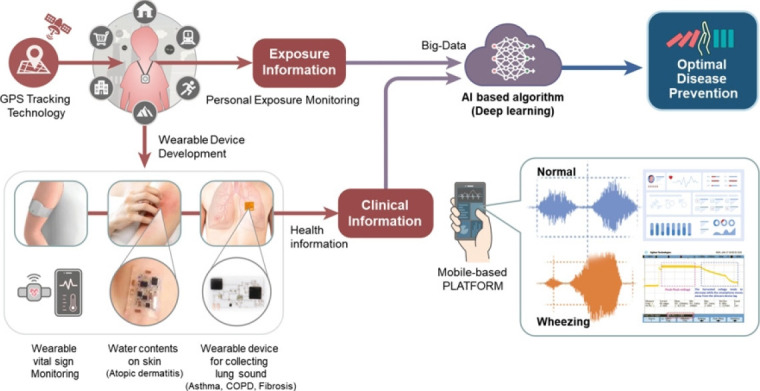
Conceptual examples of digital environmental health. GPS: global positioning system, AI: artificial intelligence; COPD: chronic obstructive pulmonary disease. Adated from the book of Ko et al. [10] with appropriate permission.

While real-time monitoring data on indoor and outdoor environmental hazards is continually being collected, there remains a significant lack of personal health data, often due to the inadequate development of wearable devices. Furthermore, technological advancements in health assessments and prevention measures for various environmental incidents have been insufficient. These incidents include respiratory diseases such as asthma resulting from the Taean oil spill and exposure to humidifier disinfectants, lung diseases associated with coronavirus exposure, and environmental disputes involving radon-emitting beds. For these reasons, future research aimed at identifying the origins of environmental hazards and related illnesses—including respiratory, circulatory, and neurological diseases—should be supported by the development of continuous, long-term health monitoring methods. These tools are essential for the real-time tracking of environmental hazards. The Ministry of Environment is currently conducting the “Children’s Birth Cohort” study to investigate the causes and health effects of environmental exposure. This extensive study requires the development of non-intrusive, user-friendly health monitoring tools that are capable of continuously evaluating and mitigating the health impacts of real-time environmental changes. These technological advancements are crucial for aiding in the investigation and assessment of the health effects of environmental hazards and for implementing preventive measures based on these findings (Figure 2 [10]).

The main requirements for proactive prevention involve minimizing exposure to environmental hazards and developing more accurate receptor-focused exposure assessment methods. To accomplish this, it is essential to generate localized, spatial, and three-dimensional (3D) building-specific concentration levels that exceed the air pollution data currently provided by the government. Additionally, refining exposure trend forecasts based on temporal patterns, especially for indoor air pollutants, is necessary. It is also crucial to implement both tangible and intangible delivery systems, such as digital service platforms, which can connect the predicted or real-time monitored levels of indoor and outdoor environmental hazards with the receptors. Generally, information technology (IT) systems of a digital platform are designed based on a completed service architecture (or framework). The architecture typically consists of both software (S/W) and hardware (H/W) components, with the H/W configuration usually derived from the S/W architecture. The S/W architecture of an IT system generally comprises (1) data generation, (2) data collection servers, (3) data analysis, (4) databases, and (5) service management.

### Digital exposure assessment

The most effective method for evaluating exposure to environmental hazards is biological monitoring, which utilizes biological samples such as urine or blood. This approach provides the most accurate measurement of exposure levels to environmental hazards through various pathways, including inhalation, skin contact, and ingestion [11]. However, the use of this method for all exposure assessments is challenging due to its invasive nature, the need to consider the half-life of substances after sample collection, and the high analytical costs involved. As a result, exposure assessments are often conducted by estimating the levels of hazards in air, water, or soil and then extrapolating these findings to human exposure. This method, however, has significant limitations. It typically involves infrequent and cross-sectional evaluations at specific time points and short sampling periods. These factors lead to substantial disparities between estimated and actual exposure levels, thereby limiting the ability to identify correlations and causal relationships between hazard exposure and health outcomes. Furthermore, because exposure assessments are usually retrospective, they do not provide information on potential future exposures, making it difficult to implement preventive measures against hazard exposure.

Exposure assessments are carried out by applying the concentration of hazards over time, taking into account the exposure pathways (refer to the equation below).


$$E=\int_{0}^{t}\;C(t)\;dt$$

Where, units are in [concentration×time]; *E*=exposure (concentration×time); *C*(t)=concentration (ppm, μg/m^3^, #/cm^3^); and *t*=time (hr, min, sec).

With adequate temporal information, it is possible to estimate more realistic exposure levels, marking the beginning of a digital exposure assessment that diverges from traditional methods. The progress of IoT and information and communications technology, which are pivotal to the fourth industrial revolution, has led to the development of devices capable of generating real-time exposure concentrations of hazardous substances. Although there are challenges in enhancing the measurement accuracy of these devices, they are highly reliable in indicating trends in hazard occurrence.

Furthermore, the relatively low cost of purchasing and operating sensor-based measurement devices, compared to traditional high-cost equipment, facilitates their widespread installation. This enables the real-time provision of exposure information and its conversion into big data, which is a significant advantage of using these low-cost sensors.

Aligning the distribution of measurement devices with individuals’ location data to estimate personal exposure levels is expected to establish the causal relationship between exposure and health outcomes. Particularly, time-based digitalized data has the potential to prevent exposures not only at the regional level but also at the individual level, thereby reducing health risks.

### Digital health information

Traditionally, health information has been measured in the human body using expensive equipment, requiring a doctor’s diagnosis in a hospital setting. The high costs and time demand associated with these traditional methods have made continuous monitoring challenging, limiting ongoing research into the correlation between environmental factors and personal health data. However, recent advancements in IoT-based miniaturization technology have facilitated the development of various wearable devices. These devices provide supplementary information that aids medical professionals in making comprehensive and objective health assessments [12]. Despite these advancements, current wearable devices still face issues such as low accuracy, large size, and challenges with wireless capabilities and battery performance. These limitations hinder the ability to collect biometric data over extended periods [12]. There is an urgent need for the development of ultra-low power, micro-sized wearable devices. Such devices would enable continuous monitoring of health changes over long periods in a preventive manner, especially for non-therapeutic purposes like monitoring the health impacts of exposure to environmental hazards [12,13].

## Conclusions: applications and future of digital environmental health

The recent shift toward a rapidly digitized environment has opened up timely opportunities for research in environmental health, particularly in the areas of prevention and cause identification [14]. By incorporating temporal and spatial information into traditionally two-dimensional data, such as the presence or concentration of hazardous substances, we can elevate this data to a 3D or higher digital format. This transformation allows us to transition from the qualitative environmental health research of the past to a more progressive, quantitative approach. Specifically, digital environmental data, which is recorded in seconds and archived continuously, can be analyzed using AI and machine learning. This analysis can predict environmental exposures and offer proactive preventive solutions.

## Environmental health research using health impact monitoring techniques

The current capacity to thoroughly track changes in patient symptoms in response to environmental factors in South Korea is limited. Technologies that allow real-time monitoring of individual environmental factors or the maintenance of symptom and observation logs are still under development. The approach to promoting technological convergence in South Korea is disjointed and spread across various ministries. The Ministry of Education, Science, and Technology supports the advancement of convergence technologies through initiatives such as the “Future Pioneer Project” (2022). Meanwhile, industrial ministries like the Ministry of Trade, Industry, and Energy focus on merging traditional industries with advanced technologies, including information technology and nanotechnology, to boost competitiveness.

In South Korea, research primarily focuses on exposure assessments and real-time measurement technologies for air pollutants such as PM_10_ and PM_2.5_. However, there are practical challenges associated with monitoring changes in indoor environmental factors. These studies often depend on statistical or meteorological modeling rather than direct sensing technologies. In contrast, the U.S. National Science Foundation (NSF) has significantly contributed to the development of micro-electro-mechanical systems (MEMS). This has been achieved by establishing centers of excellence for micromachining and by supporting student participation in conferences. Additionally, the NSF has supported research across various fields related to MEMS, including bio-environmental systems, chemical transport systems, civil and mechanical systems, design and manufacturing, electrical communication systems, and engineering education centers.

Given that vulnerable populations are more susceptible to allergies, cardiovascular diseases, and chronic illnesses, it is necessary to establish preemptive monitoring systems for key environmental disease risk factors and develop response strategies as a preliminary approach.

Wearable devices and service platforms are expected to become powerful tools in women’s health management. These technologies enable women to better understand and manage their health across various domains, such as menstrual cycle tracking, pregnancy and childbirth management, fitness, and mental health care. As technology advances, these devices and platforms are expected to provide more sophisticated and user-friendly features, heralding a new era where women can take a more proactive role in managing their health. [Fig f1-whn-2024-08-31][Fig f2-whn-2024-08-31]
